# Editorial: Chemical Sensors for Biomedical Use

**DOI:** 10.3389/fchem.2021.685563

**Published:** 2021-05-04

**Authors:** Xiaojiang Xie, Daniel Citterio, Karin Chumbimuni-Torres, Min Xue, Xudong Wang

**Affiliations:** ^1^Department of Chemistry, Southern University of Science and Technology, Shenzhen, China; ^2^Department of Applied Chemistry, Keio University, Yokohama, Japan; ^3^Department of Chemistry, University of Central Florida, Orlando, FL, United States; ^4^Department of Chemistry, University of California, Riverside, Riverside, CA, United States; ^5^Department of Chemistry, Fudan University, Shanghai, China

**Keywords:** chemical sensor, graphene, nucleic acid, BODIPY, inkjet printing

The world is constantly asking for better chemical sensors to monitor health and environment-related issues. The year 2020 in particular, has revealed the importance of the quality and availability of sensors for biomedical uses including Covid-19 related diagnosis. However, our technological development has struggled since indeed, a ready-to-use sensor product requires interdisciplinary knowledge, collaboration, and investment.

This Research Topic aimed at emphasizing chemical sensors for biomedical use. Till now, a large number of electrochemical and optical sensors have been developed to measure the levels of key species in blood (i.e., oxygen, pH, glucose, and electrolytes) (Gifford, 2013; Frost and Meyerhoff, 2015). Besides, biomolecules (e.g., DNA, proteins, and lipids) and whole cells were also targeted (Suhito et al., 2020). Electrochemical microsensors were fabricated to monitor the neurotransmitters and metabolism in tissues (Weltin et al., 2016). Generally, sensors are designed targeting important disease-related analytes, requiring small sample volume, achieving low limit of detection, with low cost and simplified operation.

This Research Topic includes 4 articles: 3 original research articles and 1 review. Ready-to-use sensors, the development of which requires interdisciplinary collaboration, face the challenges to achieve good durability, reproducibility and at the same time, high selectivity and sensitivity. Magnetic molecularly imprinted materials based on magnetic nanoparticles (mag) coated with a molecularly imprinted polymer (MIP) have attracted much attention due to their high magnetic character, chemical stability, ease of preparation, and low cost. This material has also the advantages of having high selectivity and sensitivity comparable to standard methods. Lopez et al. present the synthesis of a mag-MIP with recognition sites for amoxicillin, which was then used as a modifier in a carbon paste electrode for the detection of this analyte in milk and river water samples with almost 100% recoveries. For the functionalization of magnetic nanoparticles, the mag-MIP were prepared by a precipitation method via free radical polymerization using acrylamide as functional monomer, N,N′-methylene-*bis-*acrylamide as crosslinker, and potassium persulfate as initiator. The use of magnetic particles combined with MIP boosts the sensor surface area, making the peak current to increase, thus improving the sensitivity of the sensor. In this work, a full characterization of the magnetic nanoparticles and the MIPs is presented.

Deng et al. reported two quaternary ammonium modified BODIPY derivatives as fluorescent probes for cell imaging and metabolism research. As the BODIPY core is known to be hydrophobic, introducing the charged ammonium groups increased the water solubility. The authors went from synthesis to photophysical characterization, cell imaging and *in vivo* experiments. The results indicated that the probes show non-specific affinity for HeLa cells during live cell imaging and also exhibited non-specific affinity for subcutaneous tumor cells in mice during *in vivo* imaging.

Gao et al. reviewed the recent progress in optical sensors based on graphene and its derivatives, covering aspects related to fluorescence, graphene-based substrates for surface-enhanced Raman scattering (SERS), optical fiber biological sensors, and so on. Applications in single-cell detection, cancer diagnosis, protein and DNA sensing are discussed. Graphene and its derivatives, including graphene oxide (GO), reduced graphene oxide (RGO), and graphene quantum dots (GQDs), displayed intriguing properties, including broadband light absorption, the ability to quench fluorescence, excellent biocompatibility, and strong polarization-dependent effects, thus emerging as one of the most popular platforms for optical sensors. Exploiting the ability of GO to quench fluorescence, Kawai et al. fabricated an immunoassay microdevice on PDMS by inkjet printing, and C-reactive protein (CRP) was assayed as a proof of concept. Two reactive reagents, sulfonic acid-containing graphene oxide (SG)-antibody conjugate and fluorescently labeled CRP (30 μL each) were separately spotted at the two bottom corners of a microchannel by inkjet printing. A proper amount of trehalose was added to the two reactive reagents to allow complete dissolution of the dried spots after sample introduction. CRP was determined by a single-step competitive immunoassay (LOD 2.5 μg mL^−1^).

## Author Contributions

XX and KC-T prepared the article. DC made a revision. MX and XW did proof-reading. All authors contributed to the article and approved the submitted version.

## Conflict of Interest

The authors declare that the research was conducted in the absence of any commercial or financial relationships that could be construed as a potential conflict of interest.
